# A Cellular Automaton Model as a First Model-Based Assessment of Interacting Mechanisms for Insulin Granule Transport in Beta Cells

**DOI:** 10.3390/cells9061487

**Published:** 2020-06-18

**Authors:** Michael Müller, Mathias Glombek, Jeldrick Powitz, Dennis Brüning, Ingo Rustenbeck

**Affiliations:** 1 Institute of Dynamics and Vibrations, Technische Universität Braunschweig, D38106 Braunschweig, Germany; m.glombek@tu-braunschweig.de (M.G.); j.powitz@tu-bs.de (J.P.); 2 Institute of Pharmacology, Toxicology and Clinical Pharmacy, Technische Universität Braunschweig, D38106 Braunschweig, Germany; d.bruening@tu-bs.de

**Keywords:** simulations, cellular automaton, insulin secretion, beta cells, actin network, TIRF microscopy, experiments

## Abstract

In this paper a first model is derived and applied which describes the transport of insulin granules through the cell interior and at the membrane of a beta cell. A special role is assigned to the actin network, which significantly influences the transport. For this purpose, microscopically measured actin networks are characterized and then further ones are artificially generated. In a Cellular Automaton model, phenomenological laws for granule movement are formulated and implemented. Simulation results are compared with experiments, primarily using TIRF images and secretion rates. In this respect, good similarities are already apparent. The model is a first useful approach to describe complex granule transport processes in beta cells, and offers great potential for future extensions. Furthermore, the model can be used as a tool to validate hypotheses and associated mechanisms regarding their effect on exocytosis or other processes. For this purpose, the source code for the model is provided online.

## 1. Introduction

### 1.1. Fundamentals on the Exocytosis

Glucose is the undisputed main stimulus for the release of insulin, the main glucoregulatory hormone, from pancreatic beta cells. The beta cells respond to a ‘square wave’ glucose stimulus, either in vivo or in vitro with a biphasic pattern of insulin release, represented by a short (5–10 min) first phase, after which the secretion rate declines to about 30% of the initial peak and increases slowly again to form a second phase which can last up to several hours [1,2]. It is obvious that such a stimulation pattern is nonphysiological, but the biphasic response is the hallmark of a healthy endocrine pancreas, which becomes blurred and diminished in human type 2 diabetes or in rodent models of this disease. Typically, the loss of stimulated secretion is described as being more prominent during the first phase, but it also affects the second phase [3,4,5]. Since diminished insulin secretion can be observed in metabolically healthy groups with a high risk of future development of type 2 diabetes, it can be regarded as an independent pathogenetic factor in the development of type 2 diabetes [6,7].

Research on the mechanisms of insulin secretion has produced a plethora of data; however, our understanding of the glucose-induced biphasic pattern of insulin secretion is still incomplete. Two, not mutually exclusive hypotheses can be distinguished: (i) the insulin granules of the pancreatic beta cell consist of two or more pools with different propensities to fuse with the plasma membrane; and (ii) the increased metabolism of glucose, which is necessary for stimulated exocytosis of insulin granules, generates a complex signal pattern [8,9]. It is well established that the metabolism of glucose (and other so-called nutrient secretagogues) in beta cells increases the mitochondrial ATP generation, and thereby induces the closure of the ATP-sensitive K+ channels (KATP channels) and, in consequence, depolarizing electrical activity [10]. The depolarization-induced Ca2+ influx across the plasma membrane is the signal for the fusion of submembrane insulin granules, and has thus been termed the “triggering” signal of nutrient-stimulated insulin secretion [11]. Additionally, the mitochondrial metabolism generates signals which do not affect the electrical activity of the beta cell, and therefore, do not initiate secretion, but which increase the secretory response to nutrients [12]. These signals make up the still incompletely understood amplifying pathway [11].

This dual set of signals generated by beta cell metabolism was connected to the biphasic secretion pattern by the hypothesis that the first phase results from the fusion of membrane-docked insulin granules, whereas the second would then be formed by granules which still have to be translocated and prepared for the fusion event [13]. However, the characteristics of both phases of secretion were markedly influenced by modulating the prestimulatory conditions so as to affect the amplifying pathway [14,15,16].

The hypothesis that the biphasic kinetics of insulin secretion are due to two different pools of secretory granules seemed to be confirmed by the first measurements using total intern-reflection microscopy (TIRFM). In these investigations, the fusion rates of insulin granules which were pre-existent in the submembrane space, and those which only appeared during observation, were counted. The former could be regarded as equivalent of the readily releasable pool, while the “newcomer” granules which became more numerous during prolonged stimulation could be seen as the equivalent of the reserve pool [17,18]. The different behavior of docked and newcomer granules was explained by different Ca2+ sensitivities [19,20].

However, in later TIRF-microscopical investigations, it was found that the newcomer granules behaved either in a more stationary or a more mobile (restless) way [21]. The fusion of the latter dominated in both phases of glucose-induced insulin secretion, which led to the formulation of a revised model of insulin granule pools [22]. Finally, in contrast to earlier models of granule transport, granules do not only move towards the plasma membrane and then dock at release sites; rather, most of the granules which appear in the submembrane space return back into the cell interior after varying residence times [23,24].

The latter observations raise the question of whether the traditional view of the cortical actin web, i.e., as a barrier which separates granules of the reserve pool from those which have reached the fusion sites at the plasma membrane, is correct. This view evolved from a number of earlier observations on chromaffin cells, most notably the parallel between the disintegration of the actin ring structure, as shown in conventional epifluorescence, and the enhancement of exocytosis (see e.g., [25]). Such a parallel can be demonstrated in beta cells by the use of the actin-depolymerizing agent, latrunculin [26].

However, the traditional view that actin serves a barrier function has evolved into the hypothesis that F-actin has a dual role, i.e., both granule transport and retention in secretory cells [27]. In pancreatic beta cells, a complex of several interacting proteins anchors secretory granules within the F-actin cortex. This complex seems to act as a physical and functional scaffold, and may define a pool of releasable granules beneath the plasma membrane [28].

### 1.2. Hypotheses and Requirements for a Model

The hypothetical fundamentals described above are to be made basically verifiable in the framework of a numerical model. In this context, within the scope of the model to be created, the following processes and hypotheses are considered:

- Granules are created inside the cell.
- Granules move either diffusely or are directed, with stimuli promoting a directed movement.
- Granules can move along actin fibers, and are prevented from moving perpendicular to the strand.
- With a certain probability, granules can pass through the actin network, in cases of actin remodeling.
- Granules must exist for a certain time before they can fuse at the membrane.
- Under constant stimuli, a steady state exists for insulin secretion.

As will be explained in more detail in the discussion section, the model is also open to the use of other or even opposite hypotheses. In this paper we will first show how the simulation technique applied here can be used to understand this problem in a model-supported way.

The processes to be described are extremely complex, and are based on many local interaction mechanisms that have a significant influence on the overall behavior of the system. Due to the limited knowledge about these processes, conventional description methods (such as the finite element method or the discrete element method) are not suitable as a first model-based approach. To model systems of high complexity with locally interacting processes and state variables, the method of Cellular Automata has recently become firmly established in a variety of scientific disciplines, and will also be applied in this study. In the following section, this modeling technique is briefly introduced.

### 1.3. The Cellular Automata Method

Cellular Automata (CA) are, in principle, among the simplest modelling methods for systems with spatial and temporal dependencies. The method has its origin in computer science, where the method was compared to a “calculating machine” [29]. A formal definition of CA is provided, e.g., in [30] and [31]. According to these sources, CA comprise of four general constituents:

1. **A domain**. This is divided into discrete elements, called cells (in the informatics sense). The spatial dimension of the cells can be arbitrary, as well as their shape (or position relative to each other). Mostly two dimensional systems are considered (where the cell shape is most commonly square, triangular or hexagonal), but also numerous one-dimensional and three-dimensional systems are to be found in the literature.
2. **Inner variables of the cells**. The set of possible inner variables should be discrete and the same for all cells. The totality of the inner variables of all cells is called the “configuration”.
3. **A neighborhood convention**. Neighborhood defines which cells directly interact with each other. In most cases, this neighborhood also corresponds to a local neighborhood, specified over a certain radius. But also cells that are not locally adjacent to each other could be denoted as neighbors. The neighborhood specification is to be selected the same for all cells.
4. **The update rule**. This represents the actual dynamics of the system. The inner variable of a cell during an update step can only change on the basis of its own state and the states of its neighbors. In the research landscape, depending on the model, both parallel (i.e., all cells simultaneously) and sequential (one cell after another per step) applications are commonly used. In most applications, one update step can be associated with a certain discrete time interval.

This very abstract and open definition for a description of dynamic systems presents, at the same time, a very high degree of flexibility in terms of the application areas and complexity of the systems. Especially if partial differential equations that are suitable for systems with high complexity are not available, CA represents a very goal-oriented and pragmatic alternative to conventional modelling techniques [32]. In particular, it is the method of choice when a global system behavior is governed by local interactions that are easily describable. Therefore, CA have a wide range of applications [33,34,35]. These range from

- **System theory**, e.g., WOLFRAMs Automaton [35] or the “Game of Life” by Conway [36].
- **Sociology**, such as predator–prey systems (e.g., WaTor [37]), traffic simulations (e.g., NAGEL-SCHRECKENBERG-model [38] or pedestrian evacuation models (e.g., [39]).
- **Physics and mechanics**, e.g., the ISING model for ferromagnetism [40], recrystallization and granulation [41], crack-formation in homogeneous materials [35], Lattice-Gas-models [42] and Lattice-Boltzmann-models for fluid flow, bulk solid dynamics [43] and tribology [44,45,46].
- **Ecology, chemistry and biology,** e.g., spread of forest fires [47] or epidemics [48], chemical processes [49] and chemical reactions (e.g., [50]), biological growth of tumors [51,52] as well as patterns and fractal formation (e.g., on sea shells and animal furs [35]) and biofilm growth on tubes [53].

Notably, the applicability of CA to different kinds of transport and growth processes plays a very important role for this paper. In order to describe the granule movement that is presented in Chapter 2, the sources listed above serve as the basis for the rules used. In addition, the literature has already provided the first models to describe the granule movement at cell membranes (but mostly not in beta cells) under the influence of actin [54,55,56]. In these sources, the movement trajectories of granules were identified with the help of microscope images, and reproduced on the basis of random walk models. The probabilities used in these models correlate with the corresponding diffusion coefficients; thus, they also take into account the influence of actin. From a system theoretical or mathematical point of view, a random walk model is a special case of a Cellular Automaton, which is used in a modified and extended form in this paper.

The implementation of the aforementioned four components of a CA for this paper’s model is explained in detail in Section 2.

## 2. Modeling and Simulation

To avoid confusion in this paper regarding the term “cell” (biological cell or cell of the Cellular Automaton), the CA cell will hereafter be referred to as “element”. The model to be created here aims to provide a phenomenological description of the interactions in a beta cell that influence the transport of insulin granules and their secretion. In particular, the following effects should be considered:

- the generation of insulin granules in the center area of the beta cell
- the directed and nondirected movement of insulin granules inside the beta cell
- the directed and nondirected movement of insulin granules at the beta cell’s membrane
- the secretion at the cell membrane
- the influence of the actin network on the movement of the granules
- the influence of the aforementioned processes through the external supply of stimuli.

For the comparison between experiments and simulations, different criteria shall be used:

- the number of granules inside the cell
- the number of granules at the vicinity to the membrane
- the appearance at the vicinity to the membrane
- the character of the actin network
- insulin secretion after varying sequences of stimuli.

This implies different requirements for the model. The implementation of the required CA components in the present problem is systematically discussed below.

### 2.1. The Simulation Domain, Innner Variables and Neighboorhood

#### 2.1.1. Domain

The simulation area should represent an entire beta cell in three dimensions. In this first step, the phenomenology and feasibility of the presented concept is the focus. Therefore, the beta cell is not described with a real measured shape, but as a cube whose edge lengths of 10 µm correspond approximately to the size of a real beta cell. In order to be able to map the essential properties of the cell associated with the granules in this first approximation step, the cube is discretized into 70 × 70 × 70 equidistant elements. The size of an element thus represents, in reality, a volume of 143 × 143 × 143 nm, which corresponds approximately to the size of an insulin granule, allowing the later implementation to occur effectively. The elements at the boundary of the cube indicate the cell membrane.

#### 2.1.2. Inner Variables

Each of these 343,000 elements has exactly one of the states listed in Table 1:

The first column indicates the name of the state, and the second column its informatic implementation in the simulation program as an integer variable (“int”). In order to recognize the states in the presentations of the results in Chapter 3, they are assigned a certain color (column 3). In the later representations, the actin is shown with a red filled element, while the granules are illustrated as spheres at the respective location. It must be mentioned that actin fibers may well have smaller dimensions than the element size of 143 nm. In this context, an element with the status “actin” does not mean that at this location, the volume is completely filled with actin, but only that actin is somewhere present in this volume.

#### 2.1.3. Neighborhood

A Moore neighborhood with *n* = 1 is applied [30]. This means that all elements which share a surface, an edge or a point are in the neighborhood. Thus, every element which is not at the boundary interacts with 26 other elements.

### 2.2. Generation of Actin Networks

This study will investigate the structure of actin networks within insulin-secreting cells, and their influence on insulin granule movement and release. Since a quantitative, three-dimensional description of this network has not yet been achieved, artificially generated structures will be used, based on high resolution live cell imaging, to simulate the known relations between changes of the network and changes of insulin secretion. As a future perspective, this artificially generated actin network and its influence on the kinetics of insulin secretion may serve the purpose of identifying deficiencies in current models of insulin granule transport, and may thus stimulate further research on the role of actin and other cytoskeletal proteins in the regulation of insulin secretion.

To set up simulated actin networks and their spatial relation to insulin granules, actin fiber structures and secretory granules of insulin-secreting cells were imaged. Islets were isolated from the pancreas of NMRI mice (14–16 weeks old) by collagenase digestion, and hand-picked under a stereomicroscope. Islets were dispersed into single cells which were cultured on collagen-coated glass cover slips for up to 3 days in cell culture medium RPMI-1640 with 10% fetal bovine serum (FBS). Insulin-secreting MIN6 cells (kindly provided by Jun-Ichi Miyazaki) were seeded on glass cover slips and cultured in DMEM medium (25 mM glucose), supplemented with 6 mM L-glutamine, 10% FBS and penicillin/streptomycin. Both cell types were kept in a humidified atmosphere of 95% air and 5% CO_2_ at 37 °C.

Actin was visualized by mTagRFP-T-Lifeact-7 (provided by Michael Davidson via addgene), and the insulin granules were visualized by the cargo-directed label, insulin-EGFP [23]. Single beta cells were transduced using an adenoviral system, as described in [57]. The MIN6 cells were transfected in suspension using Lipofectamine 2000 (Invitrogen, Karlsruhe, Germany) according to the manufacturer’s protocol. TIRF microscopy or spinning disk confocal laser scanning microscopy was performed between 48 and 72 h after transfection.

#### 2.2.1. Microscope Images and Image Processing

Imaging of the primary mouse beta cells was performed by TIRF microscopy [58], and imaging of the MIN6 cells was performed by spinning disk confocal laser scanning microscopy [59,60].

The cover slip with the attached beta cells was inserted in a purpose-made perifusion chamber on the stage of an iMIC epifluorescence microscope using the L.A. software 2.4.0.17 (TILL Photonics, Gräfelfing, Germany). Fluorescence in the evanescent field was excited at 491 nm and at 561 nm. The objective was a Zeiss α-Plan-Fluar (100×, 1.45 N.A.), the angle of incidence was 68° and the calculated decay constant (reduction of the initial intensity at the glass-membrane interface to 1/e = 37%) of the evanescent field was 84 nm. After an initial equilibration period (Krebs-Ringer medium with 5 mM glucose), images were acquired; then, the perifusion chamber was filled with Krebs-Ringer medium (5 mM glucose) which contained 10 µM Latrunculin B. After 30 min of static incubation, another set of images was acquired.

**Figure 1:**
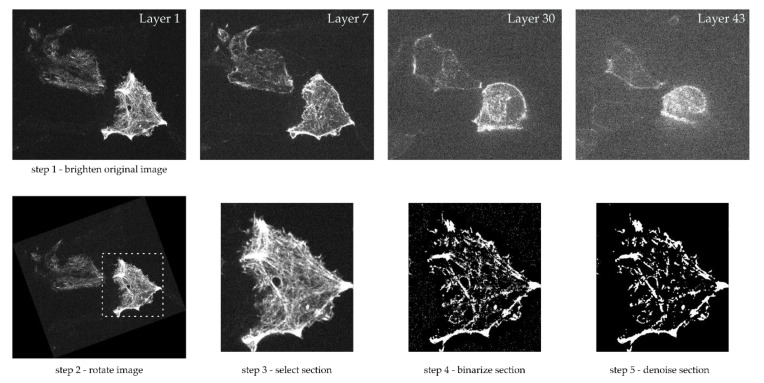
Top: Image sequence over several layers of an insulin-secreting MIN6 cell transfected with mTagRFP-T-Lifeact-7, taken with a Spinning Disk Confocal Microscope, white = actin; bottom: pretreatment image processing steps.

The cover slip with the attached MIN6 cells was pasted on a cavity glass slide filled with Krebs-Ringer medium. This glass slide was clamped upside down on the stage of an inverted Nikon Ti2-E microscope fitted with a Yokogawa CSU W1 SORA spinning disk unit. Fluorescence was excited at 561 nm and observed using a Nikon CFI SR HP Apochromat objective (100×, 1.49 N.A.). Images were acquired by a sCMOS camera (Photometrics Prime BSI, Teledyne Photometrics, Tucson, AZ, USA) and the Visiview Premier software (Visitron Systems, Munich, Germany). Images of 65 layers, each separated by a distance of 265 nm, were acquired during a static incubation under basal conditions (Krebs-Ringer medium with 3 mM glucose). An exemplary sequence of layers for a MIN6 cell is shown in the top row of Figure 1.

A MATLAB™ script was written to process these images (compare Figure 1, bottom). Since the cells on the images were not always centered and filled the image completely, the appropriate section was selected first. A rectangular format was possible, whereby a rotation of the section within the image plane was possible. Depending on the quality of the input image, the image optimization “imadjust” in MATLAB™ was used. This reassigns the intensity values, and thus, increases the contrast of the image. The image was then binarized using thresholding. We examined whether each pixel was brighter or darker than the threshold value, whereby the limit value of 50% of the maximum brightness was used. The subsequent denoising was achieved using a two-dimensional median filter. This also took into account the fact that the images not only showed the respective layer, but also partly less sharply the adjacent areas in depth direction. Thus, all actin structures are clearly visible in white, while all other cell contents are blackened. For further interpretation, the fact that the resolution in the z-direction was different from that in x- and y-direction was taken into account. While between each image, i.e., each individual plane, a distance of about 200–250 nm was present, the in-plane resolution amounted to between 20 and 60 nm per pixel.

With the help of conversion factors, the previously determined image section was scanned and an array was filled, depending on the existing structures. This array was three-dimensional and represented the real cell. Information for an x-y plane could be obtained from each image. The scanning of several images consecutively yielded information in the z- direction. The program “ParaView” [61] was used to visualize the created 3D models. This enabled the fast processing of large amounts of data in different display variations.

#### 2.2.2. Objective Characterization of Network Structures

In order to be able to objectively compare the structures of different networks, suitable measures are required to characterize the networks. These measures should also serve to generate artificial networks with adjusted properties.

The actin network within the cube is determined by elements that have the property “actin”. The most elementary integral parameter, which can describe the density of the network, is the number of elements with the property “actin” in relation to the total number of elements. This density ρ is therefore between 0 and 1, and is greater the more “actin” elements (corresponding with more actin) are in the control volume (cube).

**Figure 2:**
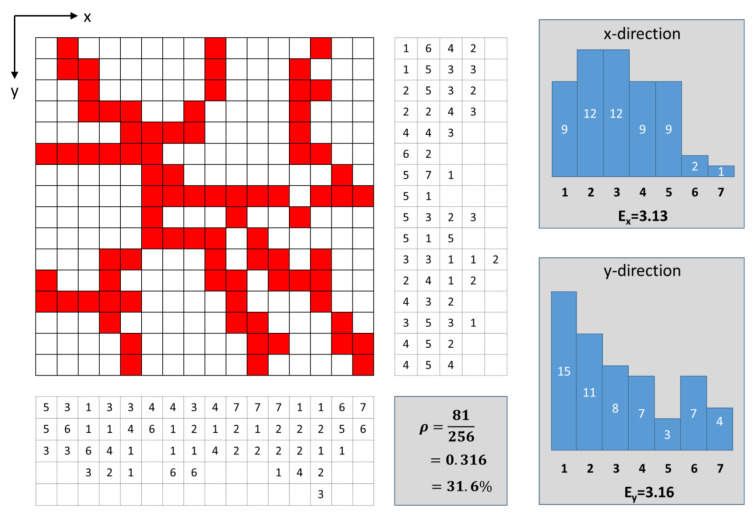
Example to explain the chord length distribution.

However, the measure of density alone is not suitable to describe the essential structure of the network. For example, the density value would not be able to distinguish whether the net consists of thick strands with a large distance between them (large mesh size) or thinner strands with a small distance between them (small mesh size). For this purpose, characterization using the so-called chord length distribution was also carried out; for further details on this strategy see [62]. Figure 2 illustrates this procedure with a two-dimensional example.

This example consists of 16 × 16 = 256 elements, of which 81 elements have the property “actin” (red). The density is therefore 31.6%. The “actin” elements are distributed in rather strand-like manner over the domain. The chord length distribution is now determined in the different directions (horizontal x-direction and vertical y-direction). For determination in the x-direction, the procedure is performed line by line, and the number of connected nonactin elements is counted until the next actin element occurs. Using the example of the first line from left to right, this means that there is first 1 “nonactin” element, then 6 “nonactin” elements behind the first “actin” element, until the next “actin” element appears. These chord lengths are now stored one after the other (see 1st row of the table to the right of the domain). This procedure is now performed separately for all lines. This creates the entire table to the right of the domain. From this table, a histogram of the frequency of the chord lengths is then generated (see histogram top right). Within this frequency distribution, the expectation value (mean value) can also be calculated, which, in this example, is 3.13 in the x- direction. The same procedure is carried out column by column in the y-direction. For each column, there is a set of chord lengths which, when applied to all columns, results in the table below the domain. A histogram can also be created from this (bottom right), from which an expectation value can be calculated (here 3.16 in y-direction).

From this distribution and the expectation value, it can now be determined whether the network has narrow meshes (then the value would be small) or wide meshes (large value). An anisotropy would also be recognizable through different values for the different directions. In the case of a three-dimensional network, it is possible to proceed accordingly, resulting in three histograms and three mean values. The objective characterization of the networks used in this paper is, therefore, based on the characteristic values “density” and “mean values of the chord lengths”, as well as the histograms of the chord length distributions. The chord lengths are usually values that are unit-related, but for many purposes, they are also related to the total size of the domain. This is also the case here, where the chord lengths are given as the number of connected elements in the respective direction, relative to the total number of elements in that direction.

**Figure 3:**
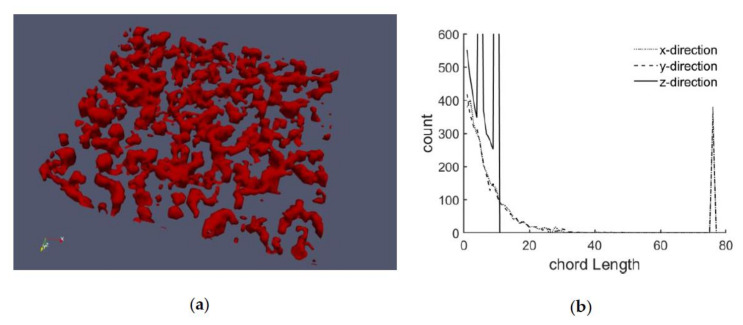
Measured actin network of a beta cell: (**a**) images of 10 layers processed to a 3D network, (**b**) chord length distributions of the network illustrated in (**a**).

Figure 3 shows a section of an actin structure measured with the Spinning Disc Confocal Microscope. For this purpose, the steps mentioned above were performed to create a 3D image (Figure 3a). Figure 3b shows the corresponding chord length distributions in the x-, y- and z- directions.

In the x and y direction, the spikes at the end represent those rows and columns that do not contain any actin. In terms of statistics, for an infinitely large grid, this peak would not exist, but with the limited number of elements, it represents the summed up value for all chord lengths greater than the number of elements in the respective direction (76). This peak is therefore ignored in the evaluation. Since only 10 layers were used for the grid in the z-direction, the peak occurs here correspondingly at a chord length of 10, and the remaining course of the curve should not be overestimated due to the relatively low number of layers.

The fact that the distribution in the x- and y-directions are almost identical indicates isotropy, or that there is no global preferred direction. The difference in the z-direction is due to the different (coarser) resolution of the images in the z-direction and the smaller number of elements (10). Since it can be assumed that in reality the distribution in z-direction is very similar to that in x- and y-direction, which could be measured with high resolution, the chord length characterization of the z-direction of the measurements will not be taken into account here. Instead, an isotropic mesh is assumed, and the characteristics from the x and y directions are adopted accordingly for the z direction.

In addition to this cell, three other cells were examined using the same method. The density as well as the mean chord lengths in relation to the total length for the respective directions are documented in Table 2.

As the table shows, the examined cells hardly differ in the value of the mean chord lengths, but rather, in density. Here, it is also evident that a higher density does not necessarily correlate with smaller chord lengths. Cells #1 and #2, as well as #3 and #4, are very similar in terms of these properties.

#### 2.2.3. Generation of an Artificial Actin Network

Various constraints are linked to the generation of an artificial actin network. In addition to the characterizing parameters explained in the previous section, these constraints relate in particular to the form and characteristics of the actin structure. This strand-like structure can therefore be most expediently created using an algorithm that generates several strands and links them together. Against this background, the basic mechanism of generation can be described as follows.

(1) The algorithm starts with an actin empty domain.
(2) In the overall area, elements are randomly selected and given the state “actin”. These serve as the starting points of the strands. They additionally receive information about the direction of the “artificial strand growth”.
(3) The next element in the direction, defined in step (2), now also becomes “actin” and retains the directional information. With a certain probability, this direction can change within a certain range. This causes the mesh to become less regular, which makes it look more natural.
(4) This procedure is carried out until the strand reaches the boundary or the limit of a randomly determined maximum length.
(5) “Reworking” is carried out, in which additional actin elements are added to existing nodes between different strands. In addition, the “thickening” of individual strands is carried out to take into account the fact that, in reality, there may also be mergers of actin fibers. In addition, individual actin elements can also be removed again, which takes into account the fact that real strands can also be interrupted or degenerate with the addition of external substances.

The parameters used in steps (2) and (5) decisively determine the density, the chord length distribution and the structure of the network. In order to achieve the given densities and chord lengths, the respective target values also serve as a basis for these parameters, which are adjusted in each step when the artificial network is created. The five listed steps do not distinguish between the directions, so the aforementioned assumption that the properties in z-direction are equivalent to those in x- and y-direction is automatically fulfilled. Although the actin network usually changes dynamically over time, it is assumed for all following considerations that it does not change, since the processes described here usually occur on a shorter time scale.

For the following simulations, four different artificial networks are used (compare Table 3):

It can be seen that all generated networks are isotropic (to be recognized by almost identical mean chord lengths in x- y- and z-directions). Network #1 is to represent the measured cells #1 and #2 respectively, network #2 is to represent the measured cells #3 and #4, and network #3 has been created to examine cells with shorter chord lengths. Network #4 represents a network that could be created by adding a cytotoxin, which would lead to a degeneration of the actin. This manifests itself in a significantly lower density and significantly longer chord lengths compared to networks #1, #2 and #3.

**Figure 4:**
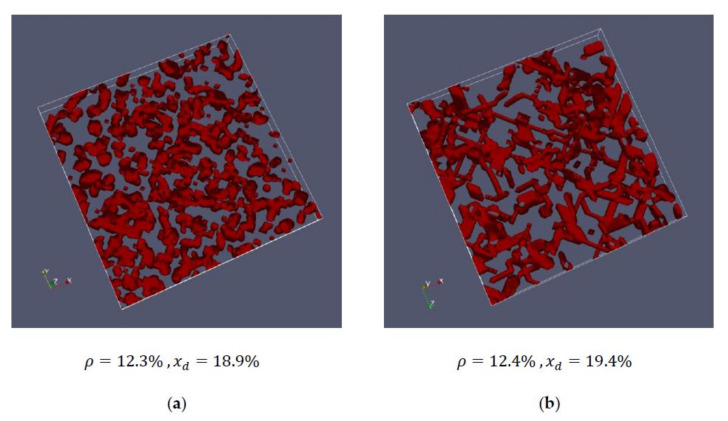
Comparison of a 10 layer actin network: (**a**) measured network of cell #1 (**b**) artificially generated network #1.

**Figure 5:**
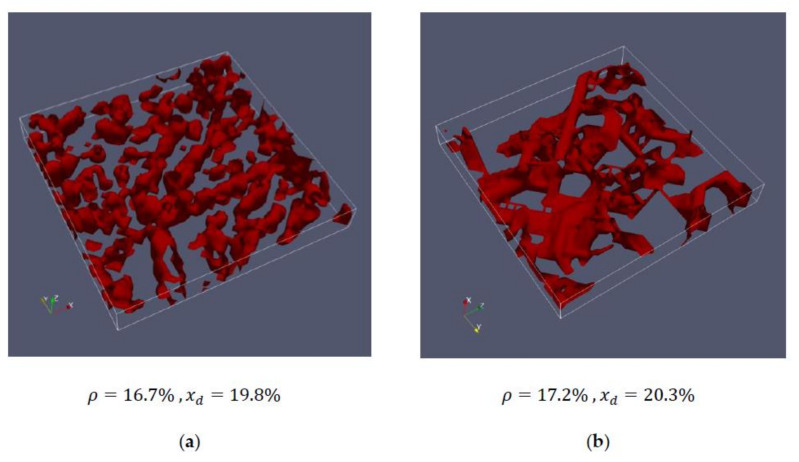
Comparison of a 10-layer actin network: (**a**) measured network of cell #4 (**b**) artificially generated network #2.

Figure 4 and Figure 5 show the comparisons between the measured cells and the corresponding artificially generated networks. In Figure 4, 10 layers of cell #1 (Figure 4a) and 10 layers of network #1 (Figure 4b) are compared, whereas Figure 5 shows 10 layers of cell #4 (Figure 5a) and network #2 (Figure 5b).

The respective networks have almost identical densities and average chord lengths, but there are still certain structural differences. This becomes particularly clear in Figure 5, where the artificial network looks more connected than the network of the measured cell. While for these first studies, the created networks represent an acceptable basis, they should be improved in the future. For this purpose, not only the mean chord length should be the same, but also the characteristic course of the entire chord length distribution.

The networks of Figure 5 have a higher density and approximately the same chord length compared to the cells in Figure 4. Here, it can be seen that the thickness of the strands is greater compared to the less dense structure in Figure 5, which is also clearly visible in the artificially generated mesh. It should be emphasized again that the thickness of the strands does not primarily mean that an actin strand has this thickness, but rather, that several actin strands lie parallel to each other to a certain extent. In the sense of the model, however, no distinction is made between them.

**Figure 6:**
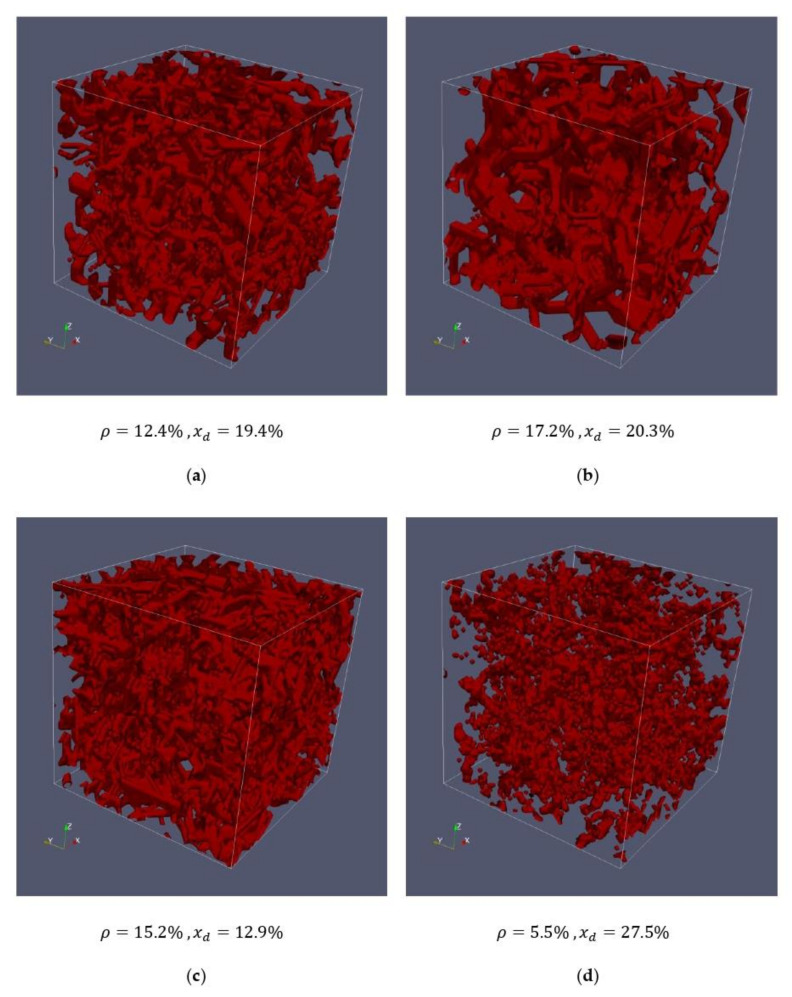
created actin networks in full size: (**a**) artificially generated network #1, (**b**) artificially generated network #2, (**c**) artificially generated network #3, (**d**) artificially generated network #4.

Figure 6 shows the artificial actin networks for the entire cell (70 layers), already characterized in Table 2 and used in the studies conducted. In addition to network #1 (Figure 6a) and network #2 (Figure 6b), which have already been compared with the measurements, networks #3 and #4 are used for comparative studies. On the one hand, Figure 6c shows a network with a similar density to networks #1 and #2, but with a significantly smaller mean chord length, which should clarify the influence of chord length on the transport of granules in the studies carried out. On the other hand, a network with a significantly lower density (Figure 6d) was generated, which should be much more permeable for granules than the other networks.

### 2.3. Set of Rules and Laws

#### 2.3.1. Calcium Concentration

The model should take into account that the cell can be stimulated in different ways. In particular, this concerns the effect of glucose and KCl stimulation. Both stimuli ultimately lead to an increase in the calcium concentration on the inner side of the cell membrane. While the influx of calcium via voltage-dependent channels plays an important role in both cases, the effect of glucose is much more complex. For example, the increased ATP generation by high glucose can activate the SERCA pumps of the endoplasmic reticulum, an effect which lowers the cytosolic calcium before it is increased by depolarization of the plasma membrane and ensuing calcium influx (for a review see [63]). With prolonged glucose stimulation complex oscillatory patterns of calcium are generated, which in addition to beta cell-intrinsic processes reflect the interaction between beta cells and neighboring beta- and nonbeta-cells contained in the pancreatic islet [64].

However, these processes usually take place at some distance from the plasma membrane and are therefore not of immediate relevance for the initiation of exocytosis, even though they may affect granule translocation from the cell interior to the plasma membrane. To keep this initial version of the cellular automaton approach reasonably simple we use just one differential equation to describe the cytosolic calcium, similar to the measurements with conventional fluorescent indicators. In the outlook section, the further steps on this point are briefly discussed.

In these terms, it is essential to consider the time offset and the different effects of glucose and KCl. Against this background, differential Equation (1) is formulated for the concentration of calcium on the inner side of the membrane.

(1) $$\frac{dC}{dt}=-\alpha \left(C-C_{S}\right)+\beta G_{0}+\gamma K_{0}$$

Table 4 explains the introduced symbols and their meaning.

Equation (1) expresses that an increase of the calcium concentration (term on the left side) is achieved by adding glucose (2nd term on the right side) or KCl (3rd term on the right side), but that this does not occur instantaneously, but with a diffusion-like delay (1st term on the right side).

The implementation of a first order differential equation with constant coefficients chosen here is, from a mathematical point of view, the simplest approach to describe the dynamics of calcium concentration under external stimulus. In [65,66], much more complex differential equations with higher order and partly variable coefficients were identified and formulated for this problem. This also results in oscillating calcium concentrations for certain cases. In the present paper, the focus is on modelling the granule movement within the cell, in which the influence of stimuli is to be considered qualitatively. In order to demonstrate the validity of this model, this simple approach for the calcium concentration is chosen, in which the number of required parameters is still manageable. However, future work will address this point in greater detail.

**Figure 7:**
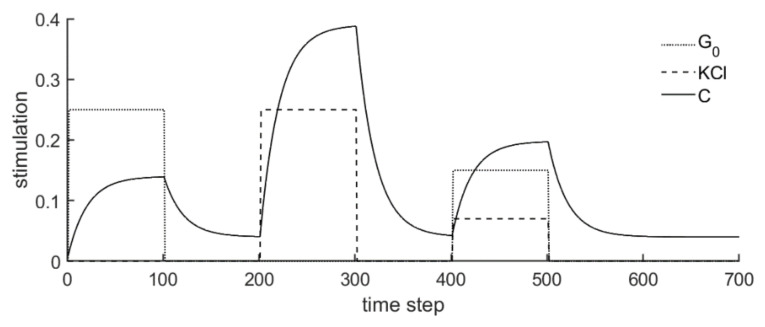
Example of the dynamic behavior of the calcium concentration on different stimuli according to Equation (1).

According to various hypotheses, the calcium concentration influences many processes of the granule movement and secretion. For this reason, the variable C is recalculated in each step by numerical integration, and then serves as an input variable for some rules of the Cellular Automaton model. One update step corresponds to a time interval of 0.1 s. If the value C is 0 at the beginning as an example (which belongs to a nonequilibrium-state), it reacts as follows, with successive stimuli by glucose ($G_{0}$) and KCl ($K_{0}$) (see Figure 7).

Here, glucose stimulation with G_0_ = 0.25 (corresponds to 25 mM glucose) is performed in the first 100 update steps (10 s); then, no stimulation is available for 100 update steps. This is followed by a KCl stimulation with K_0_ = 0.25 (corresponds to 25 mM KCl) and again a ten second (100 update steps) stimulation-free phase. Between update steps 400 and 500, KCl stimulation with 7 mM (K_0_ = 0.07) and glucose stimulation with 15 mM (G_0_ = 0.15) act simultaneously, after which there are no more stimuli (G_0_ and K_0_ are 0).

This shows that there is a delayed reaction to the respective stimuli, which rise or fall in the form of an exponential function with a negative exponent (−α). KCl stimulation naturally leads to a greater increase in calcium concentration than glucose (which is realized by γ being larger than β). Without stimulation, C always approaches the steady value $C_{S}$ (most clearly visible at the right end of the curve).

#### 2.3.2. Generation of Granules and “Age” Increase

The immediate product of translation at the rough endoplasmic reticulum is preproinsulin. Proinsulin, which results from the cleavage of the presequence, is packaged into nascent clathrin-coated secretory granules in the trans-Golgi network of the beta cell, along with other granular constituents including the proinsulin conversion enzymes [67]. From this site of generation, the granules move to the plasma membrane while undergoing several steps of maturation.

According to [68], about 13,000 insulin granules exist in a beta cell, which, on the one hand, fuse with the membrane or are degraded over time, but on the other hand, are newly produced within the cell. This results in a kind of equilibrium regarding the total number of granules in the cell. In relation to the total number of elements discretizing the entire beta cell, the total number of elements with the property to contain a granule (states 2–11) is therefore in the range $\frac{13.000}{343.000}\approx 0.038=3.8\%$.

**Figure 8:**
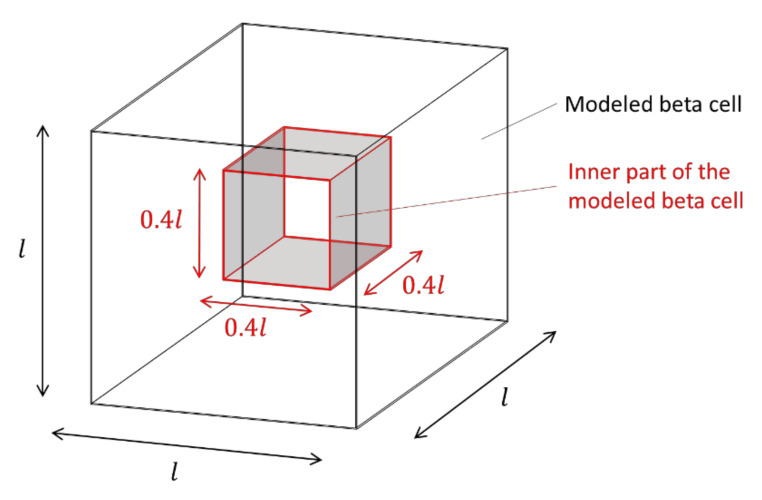
definition of the inner part of the modeled beta cell.

This fact is taken into account in the model, insofar as the generation of new granules can only take place in the inner area, which is defined here with a cube whose edge length corresponds to 40% of the edge length of the entire system (a beta cell); see also Figure 8.

For a mesh of 70 × 70 × 70 elements, the inner area thus corresponds to 28 × 28 × 28 = 21,952 elements. For the generation of new granules, first of all, the relative deviation $r_{dev}$ of the current number of granules $n_{curr}$ from the nominal number $n_{nom}$ of 13,000 is determined:

(2) $$r_{dev}=\frac{n_{nom}-n_{curr}}{n_{nom}}$$

The deviation $r_{dev}$ is a value that can theoretically be between 0 (current number $n_{curr}$ = nominal number $n_{nom}$) and 1 ($n_{curr}$ = 0). During each update step, all elements of the inner area are scanned and it is determined whether or not there is already a granule or actin in its place. If the element is still empty, a granule is created in this element with the probability of $r_{dev}$. Thus, the greater the deviation, the more granules are generated. In this way, the total number of granules is continuously adjusted to keep the nominal number of granules approximately the same. The generated granules get the state “2” (random movement) with a probability of 88% and a random state value between “5” and “10” with 12%. This correlates with the conclusions from source [69] about the probability distributions of granule movement types. In addition, for each new granule, a random value between 0 and “ripe” for the property “age” is generated and associated with the granule. The value “ripe” is set at 15 in all simulations in this study.

After each update step, the age of a granule can be increased by 1, as long as the value is less than “ripe” (maximum value). For this, the system compares whether a random number between 0 and 1 is smaller than the value a_life_*C. A large value C, and thus, a large calcium concentration, increases the frequency of an increase, which takes into account the correlation between stimuli and priming. The parameter a_life_ was set to 1 in these first studies.

#### 2.3.3. Preliminary Remarks on the Following Rules and Procedures

**Figure 9:**
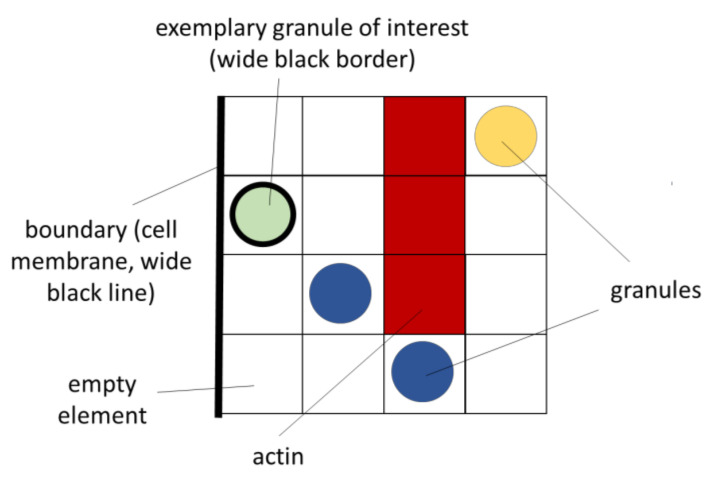
Exemplary 2D configuration for the following rules and identification of the included features.

At the core of the present model are the rules for the movement of the granules. For this purpose, special workflows have been implemented in the algorithms. These include, above all, case distinctions, i.e., whether elements are already occupied, and whether actin is at this point or, for example, the cell membrane. Furthermore, the influence of the stimuli plays a decisive role. Here, random numbers are used to decide whether a movement or change of state takes place or not. The model, thus, also has a probabilistic character. In the following paragraphs, the different rules are presented in the form of flowcharts. For a better understanding, suggestive example configurations in 2D are always integrated. To better interpret these example configurations, Figure 9 shows their essential features.

In addition, commented MATLAB™ source code is made available for the model, so that the rules can also be understood in computerized form.

#### 2.3.4. Diffuse (Random) Granule Movement (State “2”)

**Figure 10:**
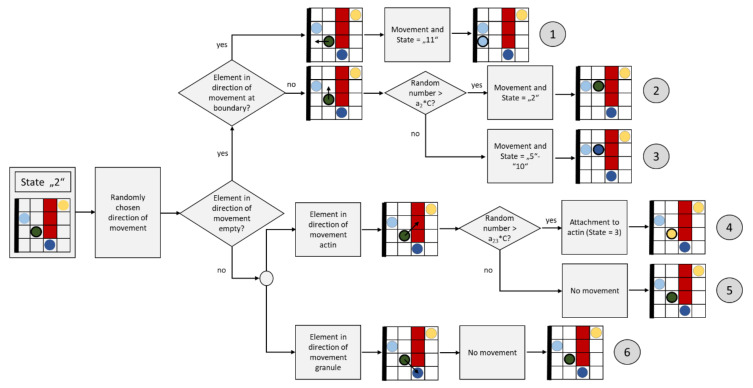
Flowchart for the update rule of a diffusely moving granule (state “2”).

Figure 10 shows the flow chart for an update step of a granule with diffuse movement (the dark green element).

With an update step, a diffusely (randomly) moving granule (element with state “2”) can move into one of its neighboring elements or remain in the current element. For this reason, one of the corresponding neighbors is selected by generating a random number where all neighboring candidates are equally probable. If this neighboring element is currently empty (state “0”), a diffuse granule (state “2”) will appear there for the next step, and the current element will be given state “0” (empty) after the update step (Figure 10, path ②). If the boundary of the domain (the cell membrane) is reached in this process, the element changes its state to “11” (granule on membrane), path ①. A diffusely moving granule can also transform its state into a directed moving granule (state “5” to “10”). The probability of such a transformation is correlated with the calcium concentration in the cell, i.e., with the variable C; the greater C is currently, the greater the probability that this transformation will occur. This is implemented by first generating a random number (evenly distributed) between 0 and 1; the greater C is, the more likely it is that this random number is smaller than a_2_*C, whereby the parameter a_2_ controls the frequency of this case. In all these first studies, a_2_ was set to 1/2, but could also take other values. If this case occurs, the granule changes its state to “5”–”10” (path ③), whereby a number between 5 and 10 is randomly chosen, which defines the subsequent direction of movement.

If the element is already populated by actin, the element remains at its position and can change its state to “3” (attaches to the actin, path ④). This change is associated with a verification of whether a random number between 0 and 1 greater than the product a_23_*C, where a_23_ = 1 was set in the simulation. A change is therefore less probable at high values of C, which takes into account the lower probability of attachment at high calcium concentrations, as implicitly (duration) postulated in [70]. If the random number is smaller, the granule remains in its state (path ⑤).

If the target element is already occupied by another granule, the granule remains in the current element and maintains its state, i.e., “2” (path⑥).

#### 2.3.5. Movement of Granules Attached to Actin (State “3”)

**Figure 11:**
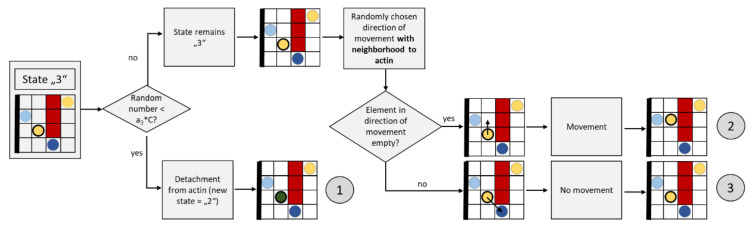
Flowchart for the update rule of a granule attached to actin (state “3”).

Figure 11 shows a flow chart for an update step of a granule attached to actin (the yellow element with wide black border).

In the case of a granule bound to actin, it is first verified whether this should remain so. Therefore, a random number between 0 and 1 is generated (evenly distributed) and it is verified whether it is smaller than a_3_*C. This occurs more frequently if the value C is high due to the stimuli. This rule takes into account the fact that a high calcium concentrations promote detachment from actin [70]. In this case, the granule changes its state from “3” to “2” (path ①).

If the granule maintains its state “3”, it can move along the actin. For this purpose, a random neighbor is searched for, which is also in the neighborhood of actin. If there is no other granule in this neighbor, the granule moves there (path ②); if the neighbor is already occupied, the granule does not move (path ③).

#### 2.3.6. Movement of Granules Fusing with the Membrane (State “4”)

**Figure 12:**
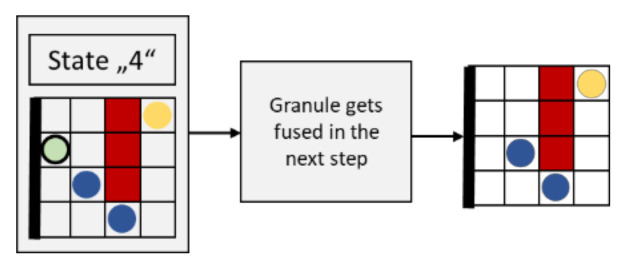
Flowchart for the update rule of a granule fusing with the membrane (state “4”).

Figure 12 shows the flow chart for an update step of a granule fusing with the membrane (the light green element with wide black border).

Each element with the state “4” fuses with the membrane in the following update step. Consequently, the state of this element changes from “4” to “0” (empty). The variable for counting fused granules is increased by 1, accordingly.

#### 2.3.7. Directed Granule Movement (State “5” to “10”)

**Figure 13:**
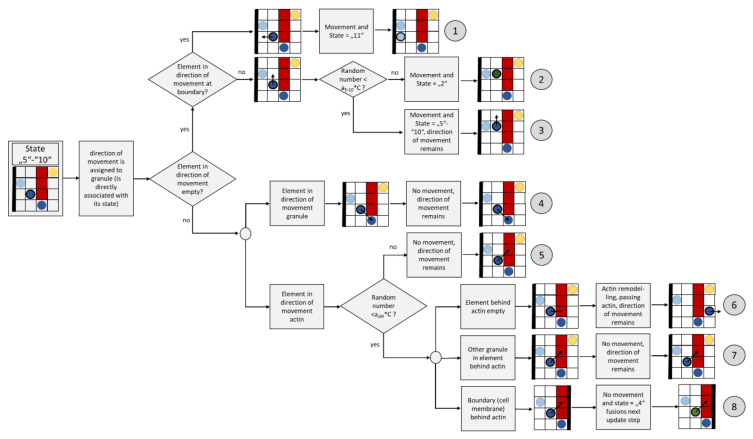
Flowchart for the update rule of a directed moving granule (states “5”–“10”).

Figure 13 shows the flow chart for an update step of a directed moving granule (the dark blue element with wide black border).

In contrast to the diffusely moving granule, the direction of movement is not randomly determined in the update step, but is fixed and does not change. Aside from that, the rules for the update step are very similar. In case the element is empty in the direction of movement (paths 1, 2 and 3), the same formulations result as for the diffuse movement (compare Section 2.3.4.). The only difference is the quantity of the parameter a_5–10_ compared to a_2_. In order to take into account the inertia of a directional motion, the reconversion from “5”–“10” to “2” is less probable than vice versa. This is implemented via a larger value a_5-10_, which is therefore set to 2.8.

If there is another granule in the direction of movement, no movement takes place (path ④). In order to take the “actin remodeling” effect into account, special rules are implemented. In the case that actin is in the direction of movement, the probability of this effect is first implemented by checking whether a random number between 0 and 1 is smaller than a_act_*C. Thus, a large value C correlates with greater probabilities of actin remodeling. The value of a_act_ is set to 1/3. If the random number is greater than a_act_*C, no actin remodeling takes place, and the granule does not move (path ⑤).

In the case that actin remodeling can take place, the state of the element behind the actin in the direction of movement is checked first. If this is empty, the granule moves there (path ⑥). If there is a granule at this position, the granule will not move (path ⑦). In case the actin is at the boundary (cell membrane), the granule changes its state to “4” and fuses in the next step (path ⑧).

#### 2.3.8. Granule Movement at the Membrane (State “11”)

**Figure 14:**
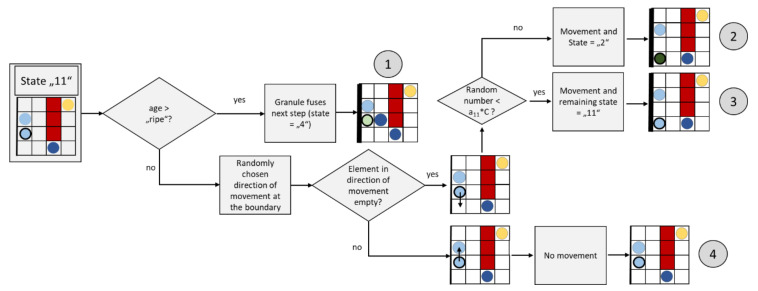
Flowchart for the update rule of a granule at the membrane (state “11”).

Figure 14 shows a flow chart for an update step of a granule moving at the membrane (the light blue element with wide black border).

A granule on the membrane whose age is already long enough (age > “ripe”) changes its state to “4” and fuses in the next update step (path ①). If the granule does not have a sufficiently long “age”, a random direction of movement along the border is determined. If the element is still free in this direction, the granule will move there. A probability check (i.e., a randomly generated number between 0 and 1 and smaller than a_11_*C?) is used to determine whether the granule retains its status “11” and remains on the membrane (path ③) or changes its status to a diffusely moving granule (path ②). This also allows granules that were already on the membrane to move back into the cell interior. A high concentration of calcium is correlated with the case whereby the granules remain on the membrane. Parameter a_11_ was set to 1 in the present simulations. If the element is occupied in the direction of movement, the granule does not move (path ④).

### 2.4. Initial Configuration, Input Variables and Output Measures

Before the simulation starts, the granules are randomly distributed in the mesh. The direction of movement can be diffuse or directional. According to [68] and [69], approximately 13,000 granules are initially distributed throughout the domain in such a way that 88% of them are given the state “2” (diffuse). This percentage is composed of 79% diffusely moving granules in the measurement and 9% nonmoving granules (since diffusely moving granules also do not move, on average, over time, this is a reasonable approach). Accordingly, 12% of the initial granules get a state of “5” to “10”, whereby the exact value is determined by a random number. At this point, it should be emphasized that a steady state is always reached, regardless of the initial configuration. Achieving this state and characterizing it is the core task of this model, and is accomplished faster, the closer the initial distribution is to the steady-state solution. The initial value of C was set to $C_{S}$, so that the system started from an equilibrium in terms of calcium concentration.

Granules docked to actin are not present at the beginning. The “age” of the elements is randomly set between 0 and the value of the variable “ripe”. About 5% of the granules are fusible; these elements thus assume an “age” that is greater by one than the variable “ripe”. With this procedure, the “Readily Releasable Pool” described in [71] and [13] is considered. Granules docked to the cell membrane are distributed at the edge of the area. About 5% of these are also considered fusible. Accordingly, granules of all movement types, as well as all fusion capabilities, are available in the network from the beginning. The distribution corresponds to a nonstimulated cell.

In addition to the given actin networks and the granules, different stimuli were implemented during the simulation, which received input into the granule movement as described above. The quantity, which plays a decisive role in the results, is the number of granules fusing per time step, which are qualitatively compared to the measured insulin secretion.

## 3. Results and Discussion

In this chapter, we will discuss the evaluation of the results generated by the Cellular Automata model. Graphs, images and information from the literature are used for comparison. At this point, it is important to point out that all generated graphs—at least in the current state of the Automaton—should only be compared qualitatively.

At first, the general functionalities of the Automaton shall be discussed in a first graphical evaluation. Afterward, the course of the insulin output in the simulation is compared with the real beta cells, and it is determined whether the simulation ends in stationary states after a certain time. Subsequently, the effects are examined by using a significantly less dense actin network, which may be the consequence of the addition of cytotoxins, for example.

Afterwards, the graphical results of the TIRFM function of the Automaton will be discussed and compared with the real TIRFM images of beta cells.

### 3.1. First Graphical Evaluation

Besides the graphs presented in the following chapters, the graphical output of the granule movement gives a good impression of the functionality of the Automaton. As already introduced, the individual granules change their state, and thus their color, in the output, depending on the stimulation.

**Figure 15:**
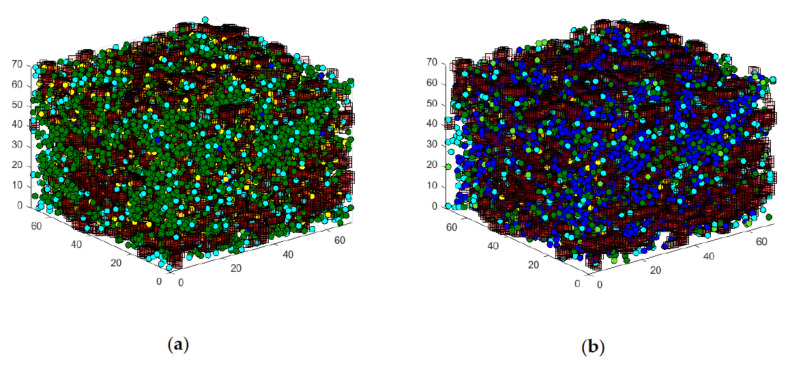
Graphical Cellular Automaton output of a configuration of the model, (**a**) without stimulation, (**b**) with glucose stimulation.

Figure 15 shows the graphical output of the state space in a comparison between case (a), without stimulation, and (b), with glucose stimulation. In the output without stimulation, the large number of green, diffusely moving granules can be seen, while the number of blue, directed moving granules is low. Furthermore, the number of yellow granules attached to the actin is relatively large. The model graphically shows, in principle, the behavior presented in Section 2.

Compared to this configuration, the increased number of directed moving granules is noticeable in the case of glucose stimulation. In addition, significantly fewer granules docked to the actin are visible. Furthermore, many fusing light blue granules are now visible at the edges of the matrix. These reflect the forthcoming insulin secretion into the cell periphery. This behavior also corresponds in principle with the processes presented in Section 2.

### 3.2. Steady State Configuration and Biphasic Behavior

**Figure 16:**
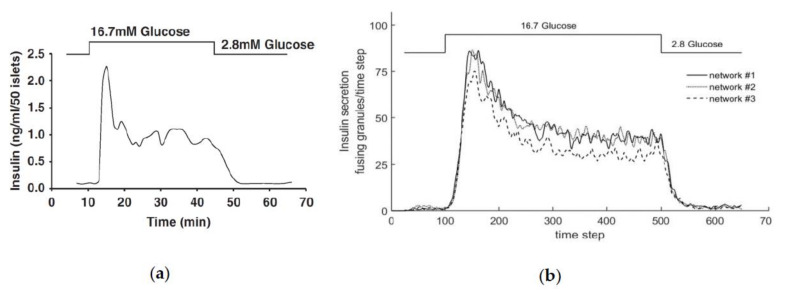
Behavior of insulin secretion at constant excitation with glucose (**a**) measured in an experiment [72], (**b**) simulated with the Cellular Automaton.

Figure 16a shows the course of insulin secretion over time, as determined in an experiment with mouse cells [72], and Figure 16b shows the insulin release in the present model over the number of iteration steps. For the values shown in the simulation, networks #1, #2 and #3 (compare Figure 6a–c) were used.

Since a qualitative comparison is carried out in the following section, primarily, the relationships to each other are compared. The graph in Figure 16a shows the insulin secretion with a stationary basic stimulation of the beta cell from 2.8 mM glucose to the tenth minute. The same stimulation is generated again from minute 45. Between the phases of the basic stimulation, glucose is supplied with a constant value of 16.7 mM. The amount of insulin secreted during the first basic stimulation is slightly above zero. About three minutes after the increase in stimulation, insulin secretion rises steeply and reaches a maximum value of 2.25 after five minutes, after which the value drops exponentially to a value of approximately 1.0, which is maintained until the start of the second phase of the basic stimulation. In phase 2, with a stimulation of 2.8 mM glucose, the amount of insulin expelled also falls to a value slightly above zero.

The glucose stimulation level and the resulting insulin release are also shown in the graph of the model (see Figure 16b). The number of granules fusing during one time step is plotted over the simulation step (which corresponds to time; 10 simulation steps are equivalent to 1 min). As before in the experiment, a small amount of glucose is supplied first, until after 100 iteration steps, which corresponds to about 10 min in the real experiment, and the value is greatly increased. The ratio between the two stimulation levels, which, in this case, are set by G_0_, was adjusted to the one used in the experiments described above. After a total of 500 iteration steps, the excitation level is restored to the low value until the simulation is finished after 650 steps.

In the model, insulin secretion begins to increase slightly for each of the networks after about 30 iteration steps, and settles at a value of about three fusions per simulation step. As the stimulation level increases, the secretion increases rapidly until it reaches its maximum after roughly 140 simulation steps. This value is held for about 30 iterations. Then, the value drops rapidly, where it remains between simulation steps 220 and 500. After the excitation level has been reduced to the basic value, insulin secretion again drops rapidly to a level of approximately three insulin fusions per simulation step.

A qualitative comparison of the graphs in Figure 16 shows very similar behavior. All graphs show two phases; the first with a maximum after increasing the excitation, and the second stationary phase at a medium secretion level. Before and after the two phases, there is a low basic secretion in both cases. The increase to the first phase begins in both cases with a slight delay, and is then very sharp.

No significant qualitative and quantitative differences in the course of the function can be observed between networks #1 and #2. Since the chord lengths of the two networks do not differ, but only the density varies, the density of the actin network does not seem to have a decisive influence on the behavior of the simulation model. In comparison, no strong qualitative difference in network #3 can be seen. However, the height of the maximum and the stationary phase are lower than in the other two networks. The chord lengths, which are smaller for network #3, therefore have an influence on the behavior of the model. The lower insulin secretion can be explained by the small movement possibilities of the granules in the network. With shorter chord lengths, smaller gaps are formed in the network, which conversely more quickly form an actin barrier for the granules. As a result, fewer vesicles reach the cell membrane simultaneously, and fewer granules fuse.

If one quantitatively compares the ratios of insulin secretion between the first and second phases of the measurement and networks #1 and #2, one obtains a value of $V_{real}=2.25/1.0=2,\;25$ for the real cell, and $V_{net\#1,2}=85/40=2,\;13$ for the simulated cells. The ratios therefore differ only slightly. Comparing the values of the first and second phase for network #3, the result is a ratio of $V_{net\#3}=73/30=2.4$, which is also very similar to that of the real cell, and again yields realistic results. Both the quantitative comparison and the qualitative analysis show that the simulation with the self-created networks represents the behavior of a real beta cell very well.

**Figure 17:**
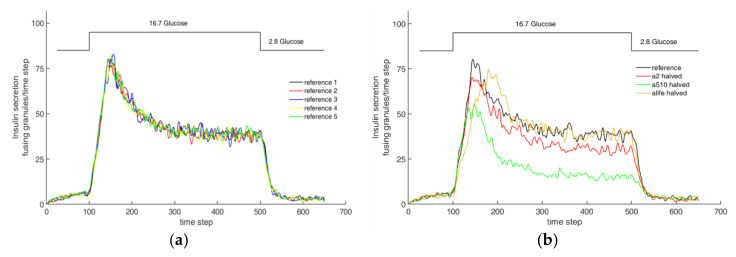
Parameter studies on the biphasic behaviour with network #1, (**a**) influence of the randomness: 5 simulations with identical parameters, (**b**) influence of some selected parameters.

Figure 17 shows the sensitivity of the randomness and system parameters to the calculated insulin secretion. Figure 17a shows the results of 5 independent simulations with identical parameters. Random variables are used in many points of the model. The difference between the curves is therefore due to the randomness of the model. However, the resulting scatter is relatively small, which can be seen from the fact that the characteristics and quantities of all curves are very similar. This is mainly due to the large number of granules in the overall model, so that the statistical noise for this question is rather small.

Figure 17b, on the other hand, represents the influence of the most important parameters a_2_, a_5-10_ and a_life_ for insulin secretion by halving these parameters one by one while fixing all other parameters. In comparison to the reference (parameters as given in the set of rules above), the halving of the parameter a_5-10_ results in a significantly smaller secretion than the reference after the granules near the membrane have fused. This is due to the fact that a small value of a_5-10_ favours diffuse movement over directional movement, which leads to a lower secretion, even in the stationary value. Similarly, the parameter a_2_ determines whether a diffusely moving granule changes into a directionally moving one. The effect is also significant, but not quite as pronounced as with a_5-10_. Another effect occurs when halving a_life_. This parameter primarily controls the rate of adjustment when calcium concentrations change. Small values of a_life_ represent a slower adjustment. However, the steady-state value is practically the same as the value for the simulation with the reference parameter set.

In summary, it can be shown that the biphasic pattern of insulin secretion can be simulated very well with the help of the Cellular Automaton, and that the parameters set within the program are appropriate. The basic behavior is realistically represented independently of the used networks, whereby the amount of insulin release is significantly influenced by the chord length, and less so by the network density; the greater the chord lengths, the higher the value of the insulin release.

Considering this result, it has to be kept in mind that the biphasic pattern of stimulated insulin secretion is typically observed with perifused islets, and is more difficult to demonstrate with single cells. However, the actin cytoskeleton reacts to the cellular environment, and may thus mediate the different insulinotropic capacities of single beta cells and beta cells within intact pancreatic islets [73].

### 3.3. Effect of An Actin Net-Degenerating Influence and Comparison with TIRF Microscopy Images

In order to investigate further the influence of actin on granule transport, latrunculin can be used, for example. This toxin, produced by marine organisms, influences the organization of the F-actin and depolymerizes the filaments [74,75]. An actin network exposed to latrunculin is, thus, significantly less dense than before. In the microtubular system, which is the stabilizer of the cell, the toxin does not cause significant changes. Thus, an otherwise unchanged functioning cell system can be assumed [76]. As a result, for a network treated with latrunculin, increased insulin output can be observed [77].

**Figure 18:**
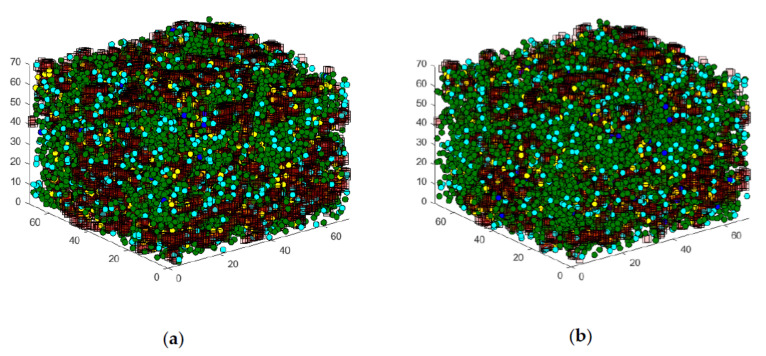
Graphical Cellular Automaton output of a configuration of the model under glucose stimulation, (**a**) with reference network #1, (**b**) with network #4, representing the cell after toxin treatment.

In the following section, the question of whether the insulin secretion of network #4 (see Figure 6d) behaves realistically will be addressed. As described above, this less dense network was created specifically to investigate the influence of a toxin on the actin structure, and thus, on granule transport. Figure 18 shows the Cellular Automaton and granules under glucose stimulation. The left illustration (a) shows the configuration for network #1, and the right illustration (b) shows the configuration for network #4. Due to the much less dense nature of actin network #4, the movement of the granules is much less hindered, and there are many more granules at the cell membrane, which leads to a greater secretion of insulin.

**Figure 19:**
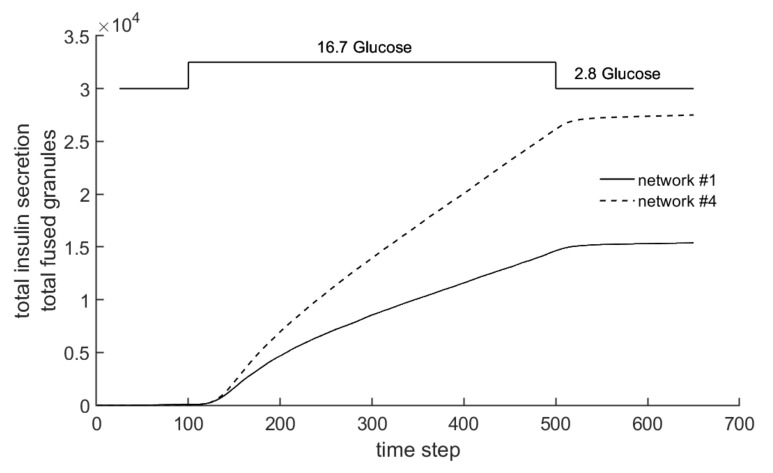
Behavior of insulin secretion of networks #1 and #4.

This effect is clarified in Figure 19. This shows the integral value of insulin secretion over the time step, which corresponds to the total amount of insulin secretion up to the respective time step. At the beginning of the stronger glucose stimulation (between time steps 100 and 140), the secretion is rather similar for the two networks. This is due to the fact that during this phase, granules from the existing Readily Releasable Pool reach the cell membrane. Later, the insulin secretion rate decreases, but in network #4, it does not decrease as much as in network #1.

Therefore, insulin secretion during the stimulation of the network supplied with latrunculin is significantly increased in relation to the original network, and is particularly characterized by a higher slope in the phase between time steps 140 and 500.

For an exemplary quantitative comparison of the insulin secretion, the values at time step 600 are considered. The relation amounts $S_{net\#4,600}/S_{net\#1,600}=\frac{27385}{15311}=1.79$, so in this case, the total secretion of network #4 is nearly 80% greater.

Although the insulin output is increased, which is triggered by the longer chord lengths, the subsequent behavior of the cell is not completely unaffected. This behavior can be explained by the interaction between long chord lengths and very low density. Since only a small amount of actin is still present, the barrier effect drops sharply. Over the course of the simulation, the granules can therefore quickly move from the production site in the cell interior to the cell membrane and fuse there.

**Figure 20:**
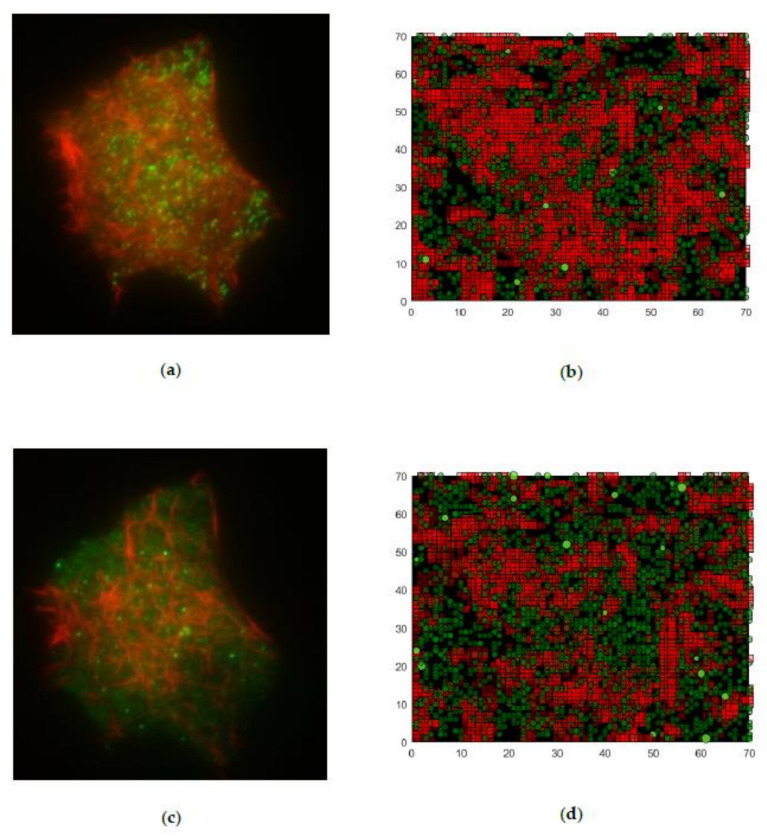
comparison between TIRF microscope pictures (left) and simulation results (right) with varying actin networks due to the supply of latrunculin, (**a**) TIRF picture of a cell before supply of latrunculin, (**b**) simulated configuration of the ten layers nearest to the boundary for network #1, (**c**) TIRF picture of a cell after supply of latrunculin, (**d**) simulated configuration of the ten layers nearest to the boundary for network #4.

Figure 20 also illustrates this characteristic. The two left figures (Figure 20a,c) show images obtained using TIRF microscopy. Figure 20a was taken on an untreated cell, and Figure 20c on a cell treated with latrunculin. Similarly, the comparisons of the simulation are shown in the right-hand area, with the first 10 layers shown in the boundary area. Therein, the elements are plotted transparently, and thus brightness decreases with distance to the boundary (as also occurs in microscopy pictures). Figure 20b shows network #1, and Figure 20d network #4. In the real cell, as well as in the simulation, it is clearly visible in the nontreated networks that actin is predominant, and only a smaller number of granules are visible. In contrast to this state, in Figure 20c,d, the granules shown in green predominate, and the actin structures are less pronounced. This is a first type of illustration that does not include a qualitative evaluation. However, it clearly shows that the trends and qualitative statements are valid.

### 3.4. Comparison with Experimental Measurements on Insulin Secretion

In [78], the behavior of the granules under different stimuli is investigated experimentally. For this purpose, beta cells of a mouse were stimulated with KCl and glucose. The results were evaluated using TIRF microscopy. The number of granules near the surface layer was examined and the amount of insulin secretion was measured.

The experiments from [78] can each be divided into five phases. The basic stimulation during the experiment is constant with 3 mM glucose. This stimulation is performed during the first, third and fifth phase of the experiments. In the two phases, an increased stimulation takes place. The following experiments were performed:

- Experiment 1: 40 mM KCl is supplied in phase 2, and 30 mM glucose in phase 4.
- Experiment 2: 30 mM glucose is supplied in phase 2, and 40 mM KCl in phase 4.

**Figure 21:**
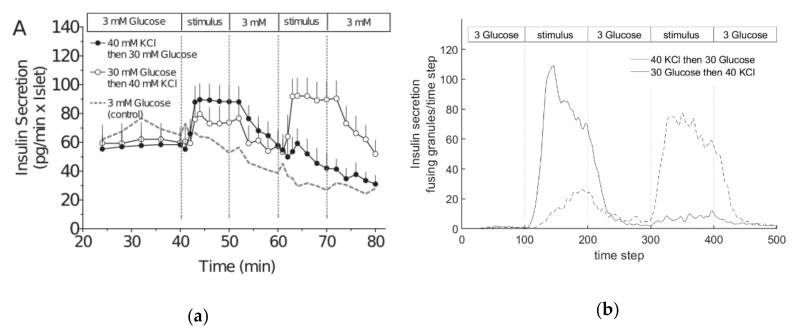
Insulin secretion when stimulated with glucose and KCl (**a**) real cell according to [78], (**b**) simulated with the Cellular Automaton model.

The evaluation of the insulin secretion of the two experiments is shown in Figure 21. Figure 21a shows the data from [78], and Figure 21b shows the curves obtained from the simulation with network #1.

The secretion of the first experiment in Figure 20a is constant during the first phase, at a value of about 60. When KCl stimulation starts, the value rises to 90 after a short delay, and remains constant until the stimulation stops again. During the following small stimulation, insulin secretion decreases approximately linearly until the subsequent stronger glucose stimulation begins. This leads to a small peak, and then to a further decreasing secretion level until the end of the experiment. In the second experiment, insulin secretion starts again at an approximate value of 60, but in the second phase, with a small delay, it only rises to 75. In the subsequent phase of low stimulation, the value drops back to the original 60. This process is repeated in phases 4 and 5, but with the KCl stimulation increasing the value to 95.

When comparing these characteristics with those from the simulation, similar behavior can be recognized qualitatively. In the simulation, the insulin output in the first experiment rises sharply with a short delay after the start of KCl supply, but in contrast to Figure 20a, falls continuously again in the further course of stimulation. During the third phase, insulin secretion again approaches zero. The second phase with increased stimulation finally produces a peak again, but this is somewhat wider than in the real cell evaluations. Finally, in the last phase, insulin secretion again tends towards zero. In the second experiment, the first increase in insulin secretion is significantly flatter than in the real cell. The subsequent decrease in phase 3, and the subsequent stronger increase in phase 4, are well illustrated. Likewise, the functional course of the simulation in the last phase falls again to a value of almost zero, with a steeper drop than in the real cell.

Overall, the results produced by the Cellular Automaton show a quite realistic course of insulin secretion. In particular, the rapid kinetics of KCl-induced secretion, generated probably by the strong influx of calcium acting on the release-ready granule pool, are clearly visible. 

In order to investigate the influence of the actin network on the experiments, Figure 21 shows all of the curves of the four generated networks simultaneously.

**Figure 22:**
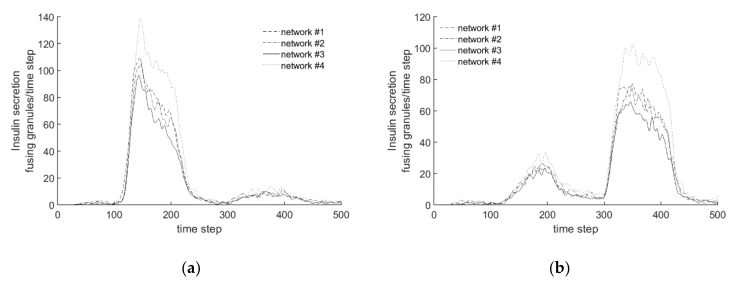
Insulin secretion when stimulated with glucose and KCl for all created networks (**a**) Experiment 1 (first KCl, then glucose), (**b**) Experiment 2 (first glucose, then KCl).

For Experiment 1, the plot in Figure 22a shows that all four courses are qualitatively similar. In the first stimulation, insulin secretion increases sharply and decreases again after the stimulation is complete. The model reacts to the subsequent second stimulation phase, with significantly lower insulin secretion in all networks. As with the stationary behavior of the model in Section 3.2., the graph of network #3 is slightly below the other graphs. The effect of the smaller chord lengths can, therefore, also be demonstrated in this experiment.

In contrast to the biphasic behavior, however, the maximum value of the function is approximately equal to the values of networks #1 and #2, but the following drop of the function is steeper. As in Section 3.2., the values of network #4 loaded with latrunculin are also greater than those of the other networks. In the peak value, the insulin secretion is higher by 1/3 compared to that of the other networks. The differences between the function courses are much more pronounced when stimulated with KCl than when glucose is supplied.

Also in the plot of Experiment 2 (Figure 22b), all graphs show a qualitatively similar course. During the first stimulation with glucose, a slight increase in insulin secretion is observed, which decreases again after stimulation. In the subsequent phase of KCl addition, there is a strong increase in the amount of fused insulin. As in Experiment 1, the amount of the peak is dependent on the networks. The courses for networks #1 and #2 with the same chord lengths but different densities do not differ significantly. Network #3, with a smaller chord length, has a slightly smaller peak, and network #4, treated with latrunculin, has a peak that is up to 25% larger. As before, the difference in the recorded functional courses is mainly due to the addition of KCl, and less to stimulation with glucose.

The comparison of the different actin networks in the simulation shows that especially the chord lengths have an influence on insulin secretion; the tighter the meshes of the network are formed, the less insulin is secreted during the experiments. In contrast, networks with the same chord length and different densities show hardly any differences in terms of secretion behavior. While the chord length influences the amount of insulin fused, the qualitative course of insulin secretion remains almost unchanged. Thus, the actin network is an essential system-determining component in addition to the processes and rules implemented in the simulation.

## 4. Conclusions

This paper presents a first approach to describing, in principle, the processes of granule transport in three dimensions and release in an insulin-secreting cell, and their dependence on the actin network in the cell. Since a multitude of biochemical mechanisms which can interact with each other are involved, a phenomenological approach was chosen to simulate secretion events. This approach, via the method of Cellular Automata, appeared to be more appropriate for this first step than conventional description methods for continua, such as finite elements or finite volumes. In spite of this conceptual simplicity, three major results were achieved:

1. The simulated pattern of insulin secretion in response to high glucose shows the typical biphasic pattern of cultured islets and cell lines [13].
2. The simulated effect of the modifier of the actin cytoskeleton, Latrunculin B, namely the increase of both phases of glucose-induced secretion, concurs with experimental observations [77], provided the simulated network fulfils a certain set of criteria.
3. This network is also compatible with a simulated interaction between glucose- and potassium-stimulated secretion, which mirrors earlier experimental observations [78].

In order to be able to perform the calculations with a suitable actin network, a 3-dimensional structure was created here using microscope images and appropriate image data processing. In order to characterize this structure and also to generate artificial actin networks, a method known from continuum mechanics was used to characterize representative volumes. With the help of the integral quantities “density” and “chord length distribution”, as well as an algorithm for the generation of actin strands, different actin networks were generated, which were oriented on the measured structures or had significantly smaller densities and larger chord lengths.

For the movement of the granules, simple phenomenological laws were formulated, with which it was effectively possible to describe the movement of the granules through the cell and at the membrane. These rules include the production of granules, their free movement, as well as their movement at the actin, and finally, the fusion. All rules are more or less based on hypotheses about the interactions which have largely been derived from experimental data. This includes, in particular, observations of granule movement in the submembrane space, which were made with TIRF microscopy.

In this context, the authors would like to emphasize that the present model represents a first step in the modelling of these complex systems. Against this background, it was initially the main objective to create a first functioning set of rules with which the user is able to model the essential phenomena and mechanisms. At the moment, the model does not yet have the property of being truly predictive, but it does offer great potential for future work.

Especially the simply structured numerics offer great advantages in computing time (the simulations documented so far took a few minutes) compared to conventional methods. Thus, on the one hand, extensions can be implemented informatically in an uncomplicated way, and on the other hand, computing capacities are not yet a problem. Rather, the addition of further findings and hypotheses is an important component in the further development of the model, so that quantitative predictions should also be possible in the future.

The dissociation of pancreatic islets into single cells and the culture of single beta cells on the artificial surface of a Petri dish will affect the organization of actin. However, this may not necessarily affect the parts of the actin network which are relevant for the granule transmission to the release sites. An additional level of complexity is given by the fact that beta cells within the islets are influenced by neighboring beta- and nonbeta-cells. So, a certain gap between the hypotheses derived from observations on single beta cells and the observable hormone release pattern of entire islets seems unavoidable. Actually, this gap may be smaller with insulin-secreting MIN6 cells, which exist primarily as single cells and can aggregate to multicellular MIN6 “pseudo-islets”.

In the next steps, the functionality of dynamically changing actin networks, as it occurs in real cells, will be integrated into the model. For this purpose, changes to the actin structure will be implemented on different time scales. On a rather short time scale, effects such as actin remodeling can be integrated in a way in which parts of the network structure are temporarily “deactivated”. On a longer time scale, it is implemented such that the network structure characteristics (such as density or chord length distribution) change over time. For this purpose, the existing algorithms for mesh generation can be used as a basis, in which the growth of a strand or its degeneration is implemented.

Further work will also consider the aspects of calcium storage in detail. Here, both more complex differential equations are to be used and a spatially resolved calcium concentration is to be considered. This also makes it possible to consider calcium stores within the cell and their interactions. The fact that the cell is already discretized in 3 dimensions in the model allows an effective implementation of the latter point.

The authors are also aware that some of the concepts of beta cell function which were used for the simulation have not yet been fully corroborated, and may evolve with further research. At the moment, our simulation serves primarily to examine effects, and thus, to test the underlying hypotheses. For this purpose, the associated software is also made available as Supplementary Materials, so that every user is also able to test his/her own hypotheses, rules and parameters.

Finally, the method of Cellular Automata is not only suitable to simulate the kinetics of insulin secretion and the preceding events, but may be a generally applicable tool to model complex processes in biological cells.

## Figures and Tables

**Table 1:** Possible states and associated colours.

State	State Int	Plot Colour	Explanation/Remark
“actin”	1	**Red**	At the element’s location, actin is present.
“granule diffuse”	2	**dark green**	At the element’s location, a diffusely (randomly) moving granule is present.
“granule actin”	3	**yellow**	At the element’s location, a granule is present that is docked to actin.
“granule fusion”	4	**light green**	At the element’s location, a granule is present that is fusing with the membrane.
“granule directed”	5–10	**dark blue**	At the element’s location, a directed moving granule is present. The moving direction can be in positive and negative direction along the x-, y- or z-axis. For these 6 possible directions, the corresponding state ints are 5, 6, 7, 8, 9 or 10.
“granule membrane”	11	**light blue**	The granule is located at the boundary (attached to the cell membrane)
“none” or “empty”	0	**transparent**	At the element’s location, none of the above states is present.
“age”	0–“ripe” + 1	-	Each granule (states 2–11) is assigned an integer value that is associated with its current lifetime (age). This (normalized) state can take a value between 0 (new) and a maximum of the value “ripe” + 1, and plays an essential role in secretion, in the sense of priming.

**Table 2:** Computed actin network densities and relative chord lengths for four cells.

Cell #	Density ρ in%	Mean Chord Lengths	
		x_d_ in %	y_d_ in %
1	12.3	18.9	19.1
2	12.1	19.1	19.5
3	16.7	17.3	17.9
4	17.6	19.8	20.1

**Table 3:** Computed actin network densities and relative chord lengths for four artificially created actin networks.

Network #	Density ρ in%	Mean Chord Lengths			Remark
		x_d_ in %	y_d_ in %	z_d_ in %	
1	12.4	19.4	17.9	18.7	corresponds to cell #1 or cell #2
2	17.2	20.3	19.9	20.2	corresponds to cell #3 or cell #4
3	15.2	12.9	13.3	13.1	network with smaller chord lengths
4	5.5	27.5	27.1	27.5	“thinned out” network

**Table 4:** Symbols in Equation (1) and their meaning.

Symbol	Meaning
C	state variable representing the calcium concentration at the inner side of the membrane
t	time (the equation’s term on the left side equals the time derivative of C)
$C_{S}$	steady state calcium concentration if no glucose or KCl is supplied; in the present studies, $C_{S}$ is set 0.04
$G_{0}$	amount of glucose (in mM/100) supplied to the cell
$K_{0}$	amount of KCl supplied (in mM/100) to the cell
α	time constant representing the time delay until a new steady state is reached after supplying a stimulus, if this value tends to zero, the time delay is large; in the present studies, α is set 0.05
β	weighting factor for the influence of the glucose supply; in the present studies, β is set 0.02
γ	weighting factor for the influence of the KCl supply; in the present studies, γ is set 0.07

## Supplementary Materials

The Sourcecode for the Cellular Automaton model are available online at https://www.mdpi.com/2073-4409/9/6/1487/s1.
