# Supplementary figures and images for: A Cellular Automaton Model as a First Model-Based Assessment of Interacting Mechanisms for Insulin Granule Transport in Beta Cells
Source: Cells. 2020 Jun 18;9(6):1487. doi: 10.3390/cells9061487 (PMC7348896; doi:10.3390/cells9061487)

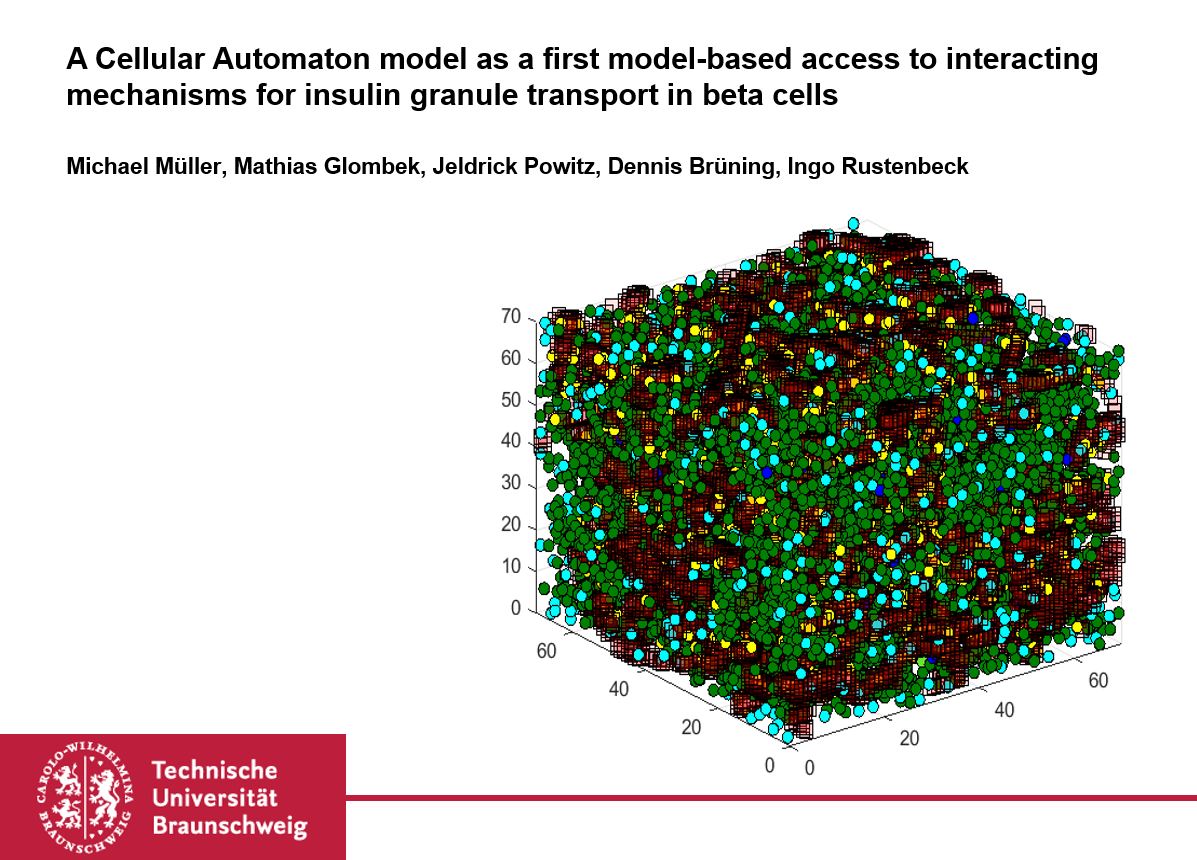

Supplement: Supplementary file 1 [file cells-09-01487-s001.zip › title_pic.JPG]
